# Covalent immobilization of VEGF on allogeneic bone through polydopamine coating to improve bone regeneration

**DOI:** 10.3389/fbioe.2022.1003677

**Published:** 2022-10-12

**Authors:** Jianhao Huang, Jingwei Lu, Ziying Liu, Jing Jin, Chunmei Xie, Yang Zheng, Zhen Wang, Lingfeng Yu, Yan Zhu, Gentao Fan, Guojing Sun, Zhihong Xu, Guangxin Zhou

**Affiliations:** ^1^ Department of Orthopedics, Jinling Hospital, The first School of Clinical Medicine, Southern Medical University, Nanjing, China; ^2^ Affiliated Jinling Hospital, School of Medicine, Nanjing University, Nanjing, China; ^3^ Nanjing Drum Tower Hospital, Nanjing, China; ^4^ Hangzhou Lancet Robotics Company Ltd, Hangzhou, China; ^5^ Nanjing Yaho Dental Clinic, Nanjing, China; ^6^ Department of Orthopaedic Surgery, Nanjing Drum Tower Hospital, Nanjing, China

**Keywords:** polydopamine (coating), surface modification, allogeneic bone, osteogenesis, angiogenesis

## Abstract

**Objective:** Promoting bone regeneration and repairing in bone defects is of great significance in clinical work. Using a simple and effective surface treatment method to enhance the osteogenic ability of existing bone scaffold is a promising method. In this article, we study the application of catecholic amino acid 3,4-dihydroxyphenylalanine (DOPA) surface coating chelated with vascular endothelial growth factor (VEGF) on allogeneic bone.

**Method:** Allogeneic bone is immersed in DOPA solution and DOPA form polydopamine (PDA) with good adhesion. Electron microscopy is used to characterize the surface characteristics of allogeneic bone. MC3T3-E1 cells were tested for biocompatibility and osteogenic signal expression. Finally, a 12-week rabbit bone defect model was established to evaluate bone regeneration capability.

**Results:** We found that the surface microenvironment of DOPA bonded allogeneic bone was similar to the natural allogeneic bone. VEGF loaded allografts exhibited satisfying biocompatibility and promoted the expression of osteogenic related signals *in vitro*. The VEGF loaded allografts healed the bone defect after 12 weeks of implantation that continuous and intact bone cortex was observed.

**Conclusion:** The PDA coating is a simple surface modification method and has mild properties and high adhesion. Meanwhile, the PDA coating can act on the surface modification of different materials. This study provides an efficient surface modification method for enhancing bone regeneration by PDA coating, which has a high potential for translational clinical applications.

## Introduction

Bone is a kind of tissue with a high potential for regeneration, and therefore most fractures or bone abnormalities can repair themselves following stable fixation (Neag et al., 2021). Mechanical stress, biochemical mediators, bioelectric and piezoelectric qualities, and neurological and endocrine impacts all influence the bone healing process (Mann and Payne, 1989). Although the above-mentioned influencing factors have been widely studied, the commonly used clinical methods are autologous bone and allogeneic bone transplantation. The autologous bone transplant is the gold standard for the treatment of bone defects because of osteogenic, histocompatible, provides structural support, and poses no danger of disease transmission (Fillingham and Jacobs, 2016). However, the use of autogenous bone transplant has a number of drawbacks, including a limited supply, an extra painful procedure to harvest bone for the graft, bleeding at the main site, varying degrees of discomfort, and wound infection (Salawu et al., 2017). Due to the abovementioned limitations, the allogeneic bone offers another option with its excellent structural support and osteogenesis. The allogeneic bone acts as a scaffold and matrix, which can simulate bone regeneration and help patients avoid extra damage (Khan et al., 2005; Fillingham and Jacobs, 2016). Despite the above advantages, the clinical application of allogeneic bone still has some shortcomings, for instance, lack of growth factors that recruit bone progenitor cells and endothelial cells, and the ability of cell adhesion (Diniz Oliveira et al., 2008; Carpena et al., 2015). Considering the abovementioned limitations, perhaps the modification of allogeneic grafts may result in better treatment effect.

Numerous surface modification strategies have been explored for improving the cell adhesion, osteogenic differentiation and vascular regeneration of allogeneic bone surfaces. Gelatin coating is one of the most commonly used methods for surface modification of allogeneic bone. The introduction of bone morphogenetic protein 2 (BMP-2) into gelatin coating could promote the adhesion and proliferation of osteoblasts. Carpena *et al.* reported that the bone scaffolds carrying gelatin and BMP-2 coating exhibited better cell viability, proliferation and cell adhesion (Carpena et al., 2015). In addition, Ring *et al.* reported the surface modification of allogeneic bone can promote vascularization and has been verified *in vivo* by double-conductive low-pressure gasplasma reactor (Ring et al., 2011). However, the gelatin coating needs to be adhered under the blow of air to maintain the hollow structure of allogeneic bone. And specialized instruments were used for surface modification of bone scaffolds. The implementation of surface modification in clinical practice is limited by complicated procedures and specialized circumstances. Future studies will focus on developing a simple and effective processing approach. Marine mussels, which are well-known for their incredible underwater sticking abilities, have gotten a lot of attention and might be a good source of tissue adhesive in the biological sector (Guo et al., 2020; Zhang X et al., 2021). The rapid and robust adhesion of mussels could be attributed to the presence of the mussel adhesive proteins, which are abundant in the DOPA (Guo et al., 2020; Zhang X et al., 2021). DOPA can self-polymerize into polydopamine (PDA) in slightly alkaline circumstances and the catechol group of PDA has excellent maneuverability during crosslinking, which it generates either covalent or noncovalent connections (Lee et al., 2007). Hu *et al.* presented a mucoadhesive film inspired by mussels that have good adherence in wet environments and good medication absorption (Hu et al., 2021). Moreover, the preparation process of mussel protein is simple, does not need special reaction conditions and instruments, and can maintain good stability at normal atmospheric temperature (Sever and Wilker, 2001). Kang et al. used a PDA treated titanium surface under alkaline conditions (pH = 8.5) to increase osseointegration and alkaline phosphatase expression *in vitro* (Kang et al., 2013). Wu *et al.* showed PDA coating to fix a small-molecule activator (LYN-1604), which has excellent osteogenesis-inducing and osteoclastogenesis-inhibiting effects (Wu et al., 2022). Michalicha et al. showed a wound dressing with good antibacterial activity was prepared by coupling PDA with antibiotics (Michalicha et al., 2021). Enhanced the scaffold’s hydrophobicity, viscosity, or impact on cellular biology by using DOPA to make modifications have been carried out and achieved ideal results (Xi et al., 2009; Qiu et al., 2018; Zhu et al., 2018; Tan et al., 2021). It is a simple and effective method to modify the surface of the allogeneic bone with PDA coating and carry drugs on the coating, which might be beneficial in the clinic.

Blood vessels are critical in the process of bone repair because they can deliver nutrients and oxygen to cells buried deep inside the tissue while also transporting waste away from the cells (Thomlinson and Gray, 1955; Grimes et al., 2014). Bone regeneration is aided by accelerating vascularization throughout the repair process (Yin S et al., 2019; Peng et al., 2020; Zhang et al., 2020; Fitzpatrick et al., 2021; Yang et al., 2021). Evidence suggests that endothelial cells and osteoblast and osteoclast lineage cells communicated with one another through molecules to induce vascularization. Type H arteries in neovascularization can actively drive the development of new bone by producing substances that encourage osteoprogenitors in the bone marrow to proliferate and differentiate (Kusumbe et al., 2014; Ramasamy et al., 2014; Xu et al., 2018; Li et al., 2021). VEGF plays a key role in the vascularization of bone tissue engineering. Endothelial cell survival, mitogenesis, migration, and differentiation, as well as vascular permeability and the release of endothelial progenitor cells from the bone marrow into the peripheral circulation are all induced by the activation of the VEGF/VEGF-receptor axis (Fahmideh et al., 2022). A potential research direction involves using VEGF to boost tissue vascularization and quicken bone rebuilding. The method of accelerating bone repair through the vascularization of implants has been studied as a feasible strategy. Kneser *et al.* successfully realized the vascularization of the solid porous matrix through arteriovenous rings, and created functional bioartificial bone tissue to reconstruct major defects by injecting osteoblasts into axial pre-vascularized matrix (Kneser et al., 2006). However, *in vitro* vascularization requires two additional operations, increasing the risk of infection. *In vivo* vascularization without additional surgery has become another direction to accelerate vascularization and bone reconstruction of defect sites. In this paper, the method of surface modification of allogeneic bone with PDA coating is helpful to accelerate vascularization and bone reconstruction after allogeneic bone implantation. The simple surface treatment may become a choice for clinicians to increase the success rate of bone rebuilding at the defect site.

This work used a mussel-inspired technique to change the surface of allogeneic bone to meet the demand for an efficient biomaterial for bone regeneration. Allogeneic bone was bathed in DOPA solution to improve allogeneic bone adherence to VEGF, which has a high cell affinity and promotes vascular regeneration. The surface microstructure was observed by scanning electron microscopy (SEM). The early adhesion and cell morphology were observed by cell fluorescence staining. The osteogenesis signal expression was observed by qPCR. And the osteogenic effect *in vivo* was observed by animal experiment. We expected that surface modified allogeneic bone would have improved osteogenic and angiogenic properties and would be useful in bone tissue engineering.

## Materials and methods

### Soaking time of DOPA

The blank 24-well plates were treated with the dopamine solution (2 mg/ml in 10 mM Tris−HCL, pH 8.5) (dopamine, Alfa Aesar, Ward Hill, United States; Tris−HCL, Beyotime, Shanghai, China) for 20 s, 40 s, 1 min, 5 min and 10 min. The 24-well plates were washed three times with PBS after being treated with DOPA. On the surface of the samples, a suspension of MC3T3-E1 cells (3,000 cells/cm^2^) was dropped. In the blank dishes, the blank group was given the same amount of MC3T3-E1. After 72 h of culturing, each group received 10 L of the cell counting kit-8 (CCK-8) solution. The optical density (OD) was measured at 450 nm after 2 h of incubation at 37°C to determine cell viability.

### Surface modification of allogeneic bone

The experiment used commercial allogeneic bone (Bio Gene, Datsing, Beijing, China). All bones were cleaned with deionized water before being submerged in dopamine solution (2 mg/ml in 10 mM TrisHCL, pH 8.5) (dopamine, Alfa Aesar, Ward Hill, United States; TrisHCL, Beyotime, Shanghai, China) for 20 s. The scaffolds were then cleaned three times with deionized water. After 3 days in 75% anhydrous ethanol, the allogeneic bone was soaked in phosphate-buffered saline (PBS, Aladdin, Shanghai, China) for 2 days. Before usage, the bones were washed three times in PBS. Direct soak was used to graft VEGF proteins (Sigma-Aldrich, St. Louis, MO, United States) onto the surface of P@Bone. To make the VP@Bone scaffolds, the P@Bone scaffolds were soaked in a VEGF solution (200 ng/ml in deionized water) under sterile conditions and shaken overnight at 4°C. The scaffolds were then cleaned three times with deionized water.

In the following sections, allogeneic bones without any coating or biomolecules are denoted the “Bone” group; Human Umbilical Vein Endothelial Cells (HUVECs) cultivated with allogeneic bones covered with PDA coating are denoted the “HP@Bone” group; HUVECs cultivated with allogeneic bones with VEGF thought the PDA coating are denoted the “HVP@Bone” group.

### Controlled release of VEGF

The VP@Bone was made using the method described above. An enzyme-linked immunosorbent assay (ELISA) was used to determine the amount of VEGF in the solution before and after immersion, as well as the loading amount of VEGF on the allogeneic bone. The bones were soaked in 2 ml PBS in an Eppendorf tube. At 37°C and 100 rpm, the tube was placed on a thermostatic oscillator (MQT-60R; Shanghai Minquan Instrument Co., Ltd., Shanghai, China). PBS was collected and frozen at 80°C at 0.5, 1, 2, 3, 5, 7, 10, 14, 20, and 30 days, and then 2 ml of additional PBS was added to the tube to continue the oscillation. The concentration was measured using the VEGF ELISA kit (KeyGEN BioTECH, Jiangsu, China) according to the manufacturer’s instructions. A spectrophotometer was used to determine the concentration (Synergy MX, Bio Tek, Winooski, United States). The cumulative amount of VEGF released at each time point was computed once the concentration was acquired, and the release curve was created.

### Surface characterization of the allogeneic bone surface modification

The surface topography was examined using a sacnning electron microscopy (SEM) (SU8020; Hitachi, Tokyo, Japan). Using a series of graded alcohols, all samples (*n* = 3 for each group) were dehydrated and dried at room temperature. For SEM, the dry samples were sputter-coated with gold–palladium and inspected with a SEM at 1 kV. At a 15 kV acceleration voltage, the energy dispersive X-ray spectroscope (EDS) linked to the FE-SEM apparatus was used to analyze the surface elements of coatings.

### Cell experiments

The Institute of Life Science Cell Culture Center (Procell Life Science and Technology Co. Ltd., Wuhan, China) provided the mouse calvarial pre-osteoblast cell line MC3T3-E1, which was cultured at 37°C with 5% CO_2_ in growth medium (GM), AMEM medium (Gibco, United States) supplemented with 10% fetal bovine serum (FBS; Gibco, United States) and 1% penicillin/streptomycin solution. The Institute of Life Science Cell Culture Center (Otwobiotect, Shenzhen, China) provided the human umbilical vein endothelial cell line HUVEC, which was cultured at 37°C with 5% CO_2_ in growth medium (GM), DMEM medium (Gibco, United States) supplemented with 10% fetal bovine serum (FBS; Gibco, United States) and 1% penicillin/streptomycin solution (P/S; Gibco, United States).

The same number of HUVECs, which were cultured in the 0.22 μm transwell, were co-cultured with the MC3T3-T1s.

### Osteogenic induction

MC3T3-E1 cells were grown in osteogenic differentiation medium (OM) (GM supplemented with 0.1 M dexamethasone (Gibco, United States), 50 g/ml ascorbic acid (Gibco, United States), and 10 mM-glycerophosphate (Gibco, United States)) at 37°C with 5% CO_2_. Every 3 days, the culture medium was changed.

### Cytotoxicity assay

MC3T3-E1 was used to perform the biocompatibility test. The processed allogeneic bones were then placed in 24-well plates, and a cell suspension (3,000 cells/cm^2^) was put onto the samples’ surface. In the blank dishes, the blank group received the same amount of MC3T3-E1. The cell counting kit-8 (CCK-8) solution was added to each group (10 μL per each group) after 3 days and 7 days of incubation. The optical density (OD) was measured at 450 nm after 2 h of incubation at 37°C, and the cell viability was estimated. After then, the cells were colored with a Calcein/PI staining test. The cells and samples were stained with calcein AM and propidium iodide (PI) for 45 min in the dark, then fixed for 30 min with paraformaldehyde. The cells were subsequently observed under a laser scanning confocal microscope (141 FV3000; Olympus).

Cell proliferation was calculated using the following equation: Cell proliferation (%) = (OD_experience_−OD_baseline_)/OD_baseline_× 100%. The absorbance of cells cultured on the dishes for the same time was taken as OD_baseline_, and the absorbance of cells cultured on the bone scaffold for the 3 days and 7 days were taken as OD_expreience_.

### Cell adhesion

To evaluate early cell adhesion, SEM and Calcein/PI staining were used. The MC3T3-E1s were passaged, and the cell suspension was applied to the samples, which were then incubated at 37°C for 2 h. Using a series of graded alcohols, samples (*n* = 3) were dehydrated and dried at room temperature. For SEM, the dry samples were sputter-coated with gold–palladium and examined at 1 kV using a scanning electron microscope. At 2 h, the cells were colored with a Calcein/PI staining to evaluate early cell adhesion. The experimental method is the same as above.

Furthermore, the MC3T3-E1s were applied to the surface of the samples, while the control group was placed on glass bottom dishes with no bone scaffolds. At 72 h, the cells were washed in PBS and fixed for 10 min in 4 percent paraformaldehyde. After being treated with 0.1 percent Triton X-100, the cells were stained with phalloidine and 4′,6-diamidino-2-phenylindole (DAPI), and the morphology of the cells was examined using a laser scanning confocal microscope. The images were analyzed by Image-J (National Institutes of Health, United States).

### ALP activity of culture medium

The cells were incubated with HVP@Bone, VP@Bone, HP@Bone, P@Bone, and blank Petri dish, respectively, after seeding in 24-well plates at a density of 3,000 cells/cm^2^ and culture at 37°C with 5% CO_2_. Every 3 days, the culture medium was replenished in half, and the incubated culture medium was stored at -20°C. The 3 days, 6 days, and 9 days culture medium were added to 96-well plates and incubated for 15 min at 37°C with 4-amino antipyrine phosphate solution from the Alkaline Phosphatase Assay Kit (Nanjing Jiancheng Bioengineering Institute, China), followed by the addition of the stop solution. At 520 nm, the absorbance was measured using a microplate reader.

### Real-time RT-PCR

The RNA-Quick Purification Kit (Yishan Biotech, Shanghai, China) was used to isolate total RNA from differentiated cells for 7 days, and cDNA was generated with a HiScriptIIQ RT SuperMix for qPCR (Vazyme Biotech, Nanjing, China), followed by analysis with a ChamQTM SYBR Color qPCR Master Mix (Vazyme Biotech, Nanjing, China) (Vazyme Biotech). A LightCycler 480-II was used for amplification and detection (Roche, Mannheim, Germany). Supplementary Table S1 contains a list of primers. The target transcript’s levels were compared to those of the internal reference (2−ΔΔCT method).

### Animal experiments

All animal research was conducted in compliance with the regulations and procedures of the Drum Tower Hospital Affiliated to Nanjing University’s Medical School (Nanjing, China), as well as the recommendations of the Institutional Animal Care and Use Committee (IACUC).

In the current investigation, 30 male New Zealand white rabbits weighing 2.5 kg were randomly separated into five groups (HVP@Bone, HP@Bone, VP@Bone, Bone, and Blank groups; *n* = 6 rabbits per group). The HVP@Bone, and HP@Bone group were co-cultured with HUVECs for 3 days before implantation. The surgery was carried out under general anesthesia with a 0.2 ml/kg intramuscular injection of xylazine hydrochloride (Jilin Huamu Animal Health Products Co., Ltd., Jilin, China). Knee surgery was performed in a sterile environment. The lateral femoral condyle was exposed after a lateral parapatellar incision was created. A defect with a depth of 8 mm and a diameter of 5 mm was created and filled with the prepared bone of similar size (weight: 0.1 ± 0.005 g). The bone defect of the control group did not place anything and the skin was closed using 1–0 nylon sutures. The rabbits were returned to their cages after surgery and allowed to resume full weight-bearing activities. Penicillin was given intramuscularly for 3 days after surgery to avoid infection. All animals were slain 12 weeks following surgery to test bone repair.

### Micro-CT and 3D reconstruction

The harvested femurs were examined on a viva CT 80 system (V6.5–3 Scanco Medical, Bruettisellen, Switzerland), with the following operation parameters: 72 keV; 55 μA; FOV, 32 mm; integration time, 200 ms. MIMICS 19.0 was used to recreate the 3D models of the harvested femurs (Materialise, Leuven, Belgium). The software imported micro-CT scanned images and segmented them based on the range of grayscale values. Determine the ratio of the bone tissue volume (BV/TV), trabecular thickness (Tb.Th), trabecular number (Tb.N), and trabecular separation (Tb.Sp) (*n* = 6).

### Histological analysis

The collected femurs were then cleaned with deionized water, paraffin embedded, and sectioned at 5 m after being fixed with formalin at 4°C for 24 h and decalcified with 10 percent formic acid (Macklin, Shanghai, China) for 28 days. Hematoxylin and eosin (H&E), Masson’s trichrome, type I collagen and α-SAM stain were used to stain the sections, which were then examined under a microscope with a CCD camera (Olympus, Japan). The images were analyzed by Image-J (National Institutes of Health, United States).

### Statistical analysis

Three researchers evaluated the macroscopic and histological data while being blinded to categorization. For statistical analysis and exponential curve fitting, IGOR Pro 6.12 and SPSS 19.0 (IBM Corp., Armonk, NY, United States) software were employed (WaveMetrics Inc., Portland, OR, United States). Unpaired Student’s *t*-test was used to examine the findings, which were presented as mean and standard deviation. A *p* value of less than 0.05 was used to determine statistical significance.

## Results

### Soaking time of DOPA

MC3T3-T1s were cultured on the different time-processed dish for 72 h and cell proliferation was tested using CCK-8. As shown in Supplementary Figure S1, the cell proliferation between groups treated with different DOPA soaking time was no different. The DOPA treatment time of 20 s was selected as the subsequent experimental condition.

### Characterization of the VP@Bone

**FIGURE 1:**
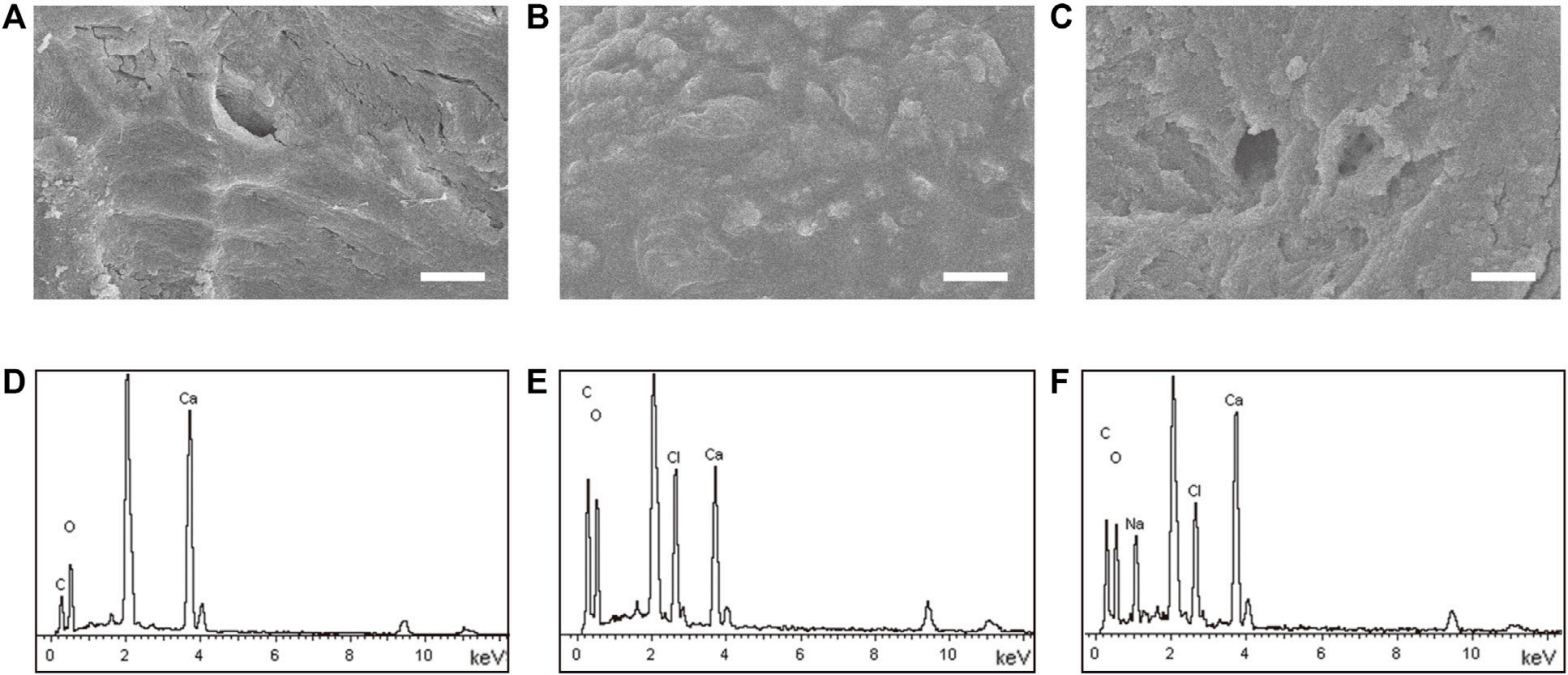
The result of the SEM images and the surface element. The SEM images of the allogeneic bone **(A)**, the P@Bone **(B)** and VP@Bone **(C)** (scale bar: 10 μm). Surface element of the allogeneic bone **(E)**, the P@Bone **(F)** and VP@Bone **(G)**.

As shown in Supplementary Figure S2, untreated allogeneic bone was a white trabecular hollow structure. The allogeneic bone soaked in DOPA solution and adhered to VEGF was black, and its shape and structure were not changed. SEM showed the surface of allogeneic bone was rough and cracked. A few protuberances could be seen on the surface of P@Bone and no obvious changes were found on the surface of VP@Bone (Figures 1A–C). The P@Bone group and VP@Bone group had a similar content of the surface element to the allogeneic bone, including carbon, oxygen, and calcium. The content of chlorime in the P@Bone group and VP@Bone group were more than the allogeneic bone, which cause by DOPA solution and was not obvious toxicity to cells (Figures 1D–F and Table 1).

**TABLE 1:** Different elements on the surface of the Bone, P@Bone and VP@Bone scaffolds examined by EDS.

	Bone (%)	P@Bone (%)	VP@Bone (%)
C	13.28	26.92	34.94
O	52.36	48.96	36.12
Ca	34.36	23.28	15.80

**FIGURE 2:**
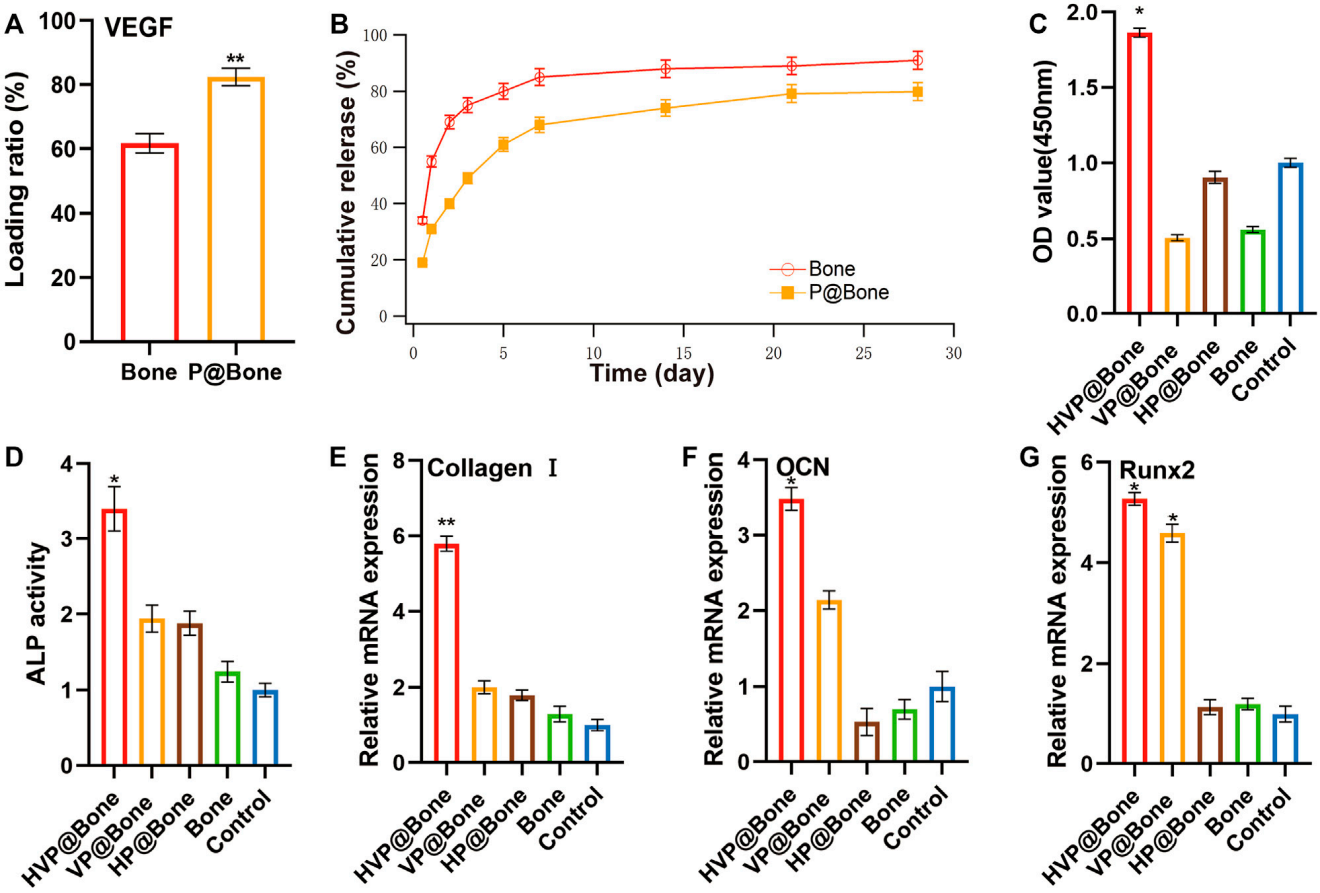
**(A–B)** Loading ratio and release profiles of VEGF in Bone or VP@Bone. **(C)** The relative cell vitality after culturing for 7 days **(D)** The culture medium ALP activity at 9 days. **(E–G)** The RT-qPCR of Collage I, OCN and Runx2 at 7 days (*n* = 3). (ns indicates no significant differences; * indicates significant differences, *p* < 0.05; ** indicates highly significant differences, *p* < 0.01; *** indicates highly significant differences, *p* < 0.001).

The allogeneic bone adhered to DOPA solution was immersed in 200 ng/ml VEGF solution and refrigerated at 4°C overnight, which enabled VEGF to adhere to the allogeneic bone. An ELISA was used to further evaluate the function of the PDA coating. The results revealed that the amount of VEGF was 164.80 (±4.45) ng in the P@Bone group after incubation. The amount of VEGF was 123.40 (±3.70) ng in the Bone group, respectively (Figure 2A). Thus, the DOPA coating could improve the loading capacity of the growth factors by more than 20%. The cumulative release curve (Figure 2B) showed that there was a gentle release with 31.71% of the total VEGF released from the VP@Bone on the first day, and the release was subsequently slowed down with approximately 68.80% of the total release of VEGF after 7 days. The sustained release could still be observed afterward. In contrast, burst releases with 55.02% of the total VEGF were identified in the Bone group for the first day and almost 85.17% of the total VEGF were released after 7 days. These results indicate that the absolute content of VEGF adhesion was increased and controlled release of the VEGF could be achieved with the help of the PDA coating.

### Biocompatibility test

**FIGURE 3:**
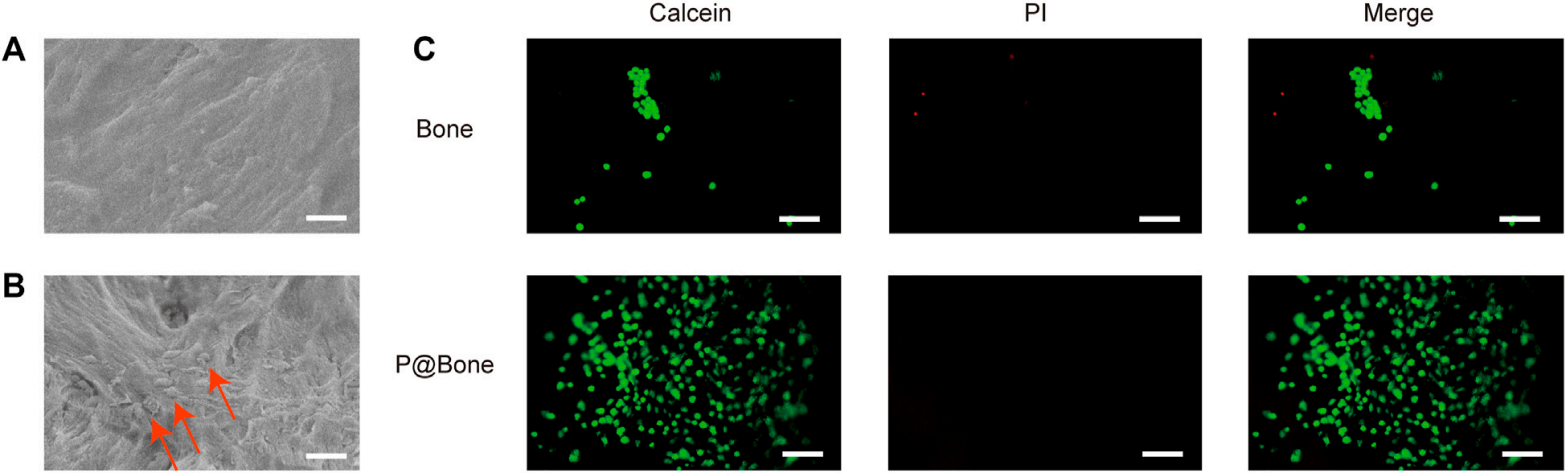
Early MC3T3-T1 cells adhesion. The SEM images of the allogeneic bone **(A)** and the P@Bone **(B)** at 2 h after cell planting (scale bar: 10 μm). The cells were marked with arrow. The result of Calcein/PI staining at 2 h after cell planting (scale bar: 300 μm).

MC3T3-T1s were cultured on the surface of various allogeneic bones at the same initial concentration to assess the biocompatibility of the bone. In early adhesion experiments, MC3T3-T1s were implanted on the surface of bone scaffolds. The SAM results showed that there was more cell adhesion on the surface of P@Bone group than on the surface of Bone group (Figures 3A,B). Meanwhile, Calcein/PI staining showed similar results. MC3T3-T1s could better adhere to the bone surface with the help of the DOPA coating (Figure 3C). ImageJ software was used to semi-quantify the fluorescence results. The average fluorescence intensity of P@Bone group was 485.17 (±32.54), which was much larger than the 0.07 (±0.01) of the Bone group (Supplementary Figure S3).

**FIGURE 4:**
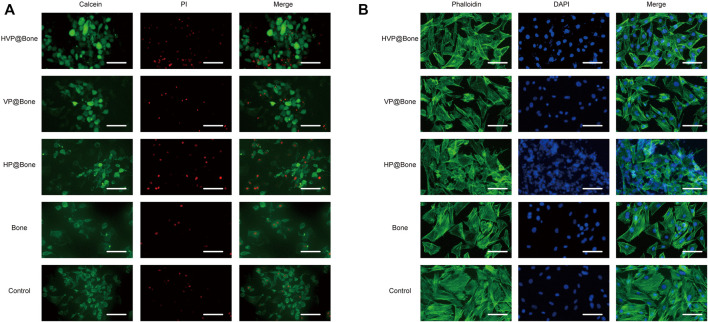
**(A)** The results of Calcein/PI staining at 3 days (scale bar: 100 μm). **(B)** The results of Phalloidine/DAPI staining at 3 days (scale bar: 100 μm).

The cell biocompatibility was evaluated by the Calcein/PI staining and Phalloidine/DAPI staining for 72 h. The Calcein/PI staining results showed no obvious cytotoxicity in each group, and the proportion of living and dead cells was similar (Figure 4A). The Phalloidine/DAPI staining results showed good biocompatibility in HVP@Bone group, in which the fully extended cytoskeleton can be seen by staining (Figure 4B). According to the result of CCK-8, the cell proliferation of HVP@Bone group was higher than the control group at 7 days (Figure 2C, Supplementary Figure S4).

### *In vitro* osteogenic study

The ALP activity is an important indicator of osteogenic differentiation. ALP activity assay was used to evaluate the ability to induce osteogenic differentiation of MC3T3-T1s at different stages. The result showed that the ALP activity of HVP@Bone group was significantly higher than the control group at 9 days (Figure 2D, Supplementary Figure S5–6). More generally, the VP@Bone and HP@Bone group exhibited increasing trends compared with the control group, indicating VEGF may play an important role in the osteogenic signal expression of MC3T3-T1s.

RT-qPCR was used to determine the ability of the bones in each group to induce the ontogenetic differentiation of MC3T3-T1s. It was shown that, the HVP@Bone and VP@Bone groups had impressive expression levels of Collagen Ⅰ, OCN and Runx2 at days 7 (Figures 2E–G). The sustained release of VEGF in the scaffold continuously induced the osteogenic differentiation of stem cells inside and outside the scaffold, which had the better potential for repairing bone defects.

### Micro-computed tomography scanning and analysis

**FIGURE 5:**
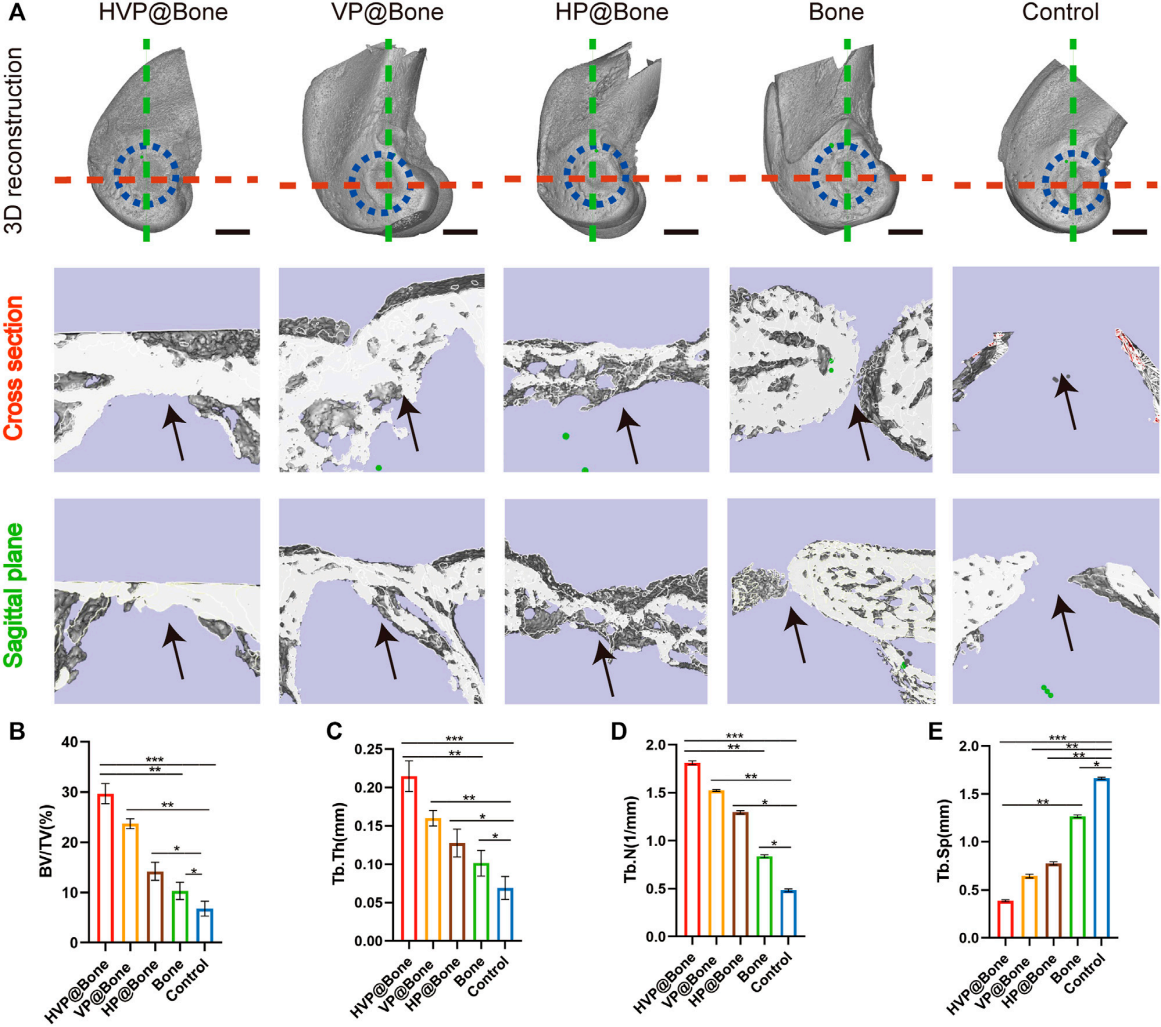
*In vivo* osteogenic effects of the different groups. **(A)**Reconstruction of 3D micro-CT images and 3D reconstruction of cross section and sagittal plane of defect area. The black arrow indicates the area of bone regeneration. (scale bar: 5 mm). Quantitative analysis of micro-CT images after 8 weeks of respective scaffolds implantation, including BV/TV **(B)**, Tb.Th **(C)**, Tb.N **(D)**, and Tb.Sp **(E)**.

Micro-CT was used to scan rabbit femur specimens to observe the repair of bone defects. Only a modest quantity of new bone tissue was generated in the control group 12 weeks following the procedure, as seen from the perspective of the 3D reconstruction of the defect site. In comparison to the control group, there were more new bone tissues generated in the HVP@Bone, VP@Bone, HP@Bone, and Bone groups. Among them, the HVP@Bone group had the most bone tissue at the distal femoral defect and the maximum repair area (Figure 5A). To further quantify the new bone tissue, the proportion of BV/TV (Figure 5B), Tb.Th (Figure 5C), Tb.N (Figure 5D), and Tb.Sp (Figure 5E) were calculated. The BV/TV, Tb.Th and Tb.N of HVP@Bone group, VP@Bone group,HP@Bone group and Bone group were significantly (*p* < 0.05) higher than those of the control group, respectively. And the Tb.Sp of HVP@Bone group, VP@Bone group, HP@Bone group and Bone group were significantly (*p* < 0.05) lower than the control group. At the same time, the HVP@Bone group observed increased complete bone cortex compared with the Bone group under Micro-CT. The HVP@Bone group showed better bone tissue regeneration compared with the Bone group.

### Histological examination of rabbit femurs

**FIGURE 6:**
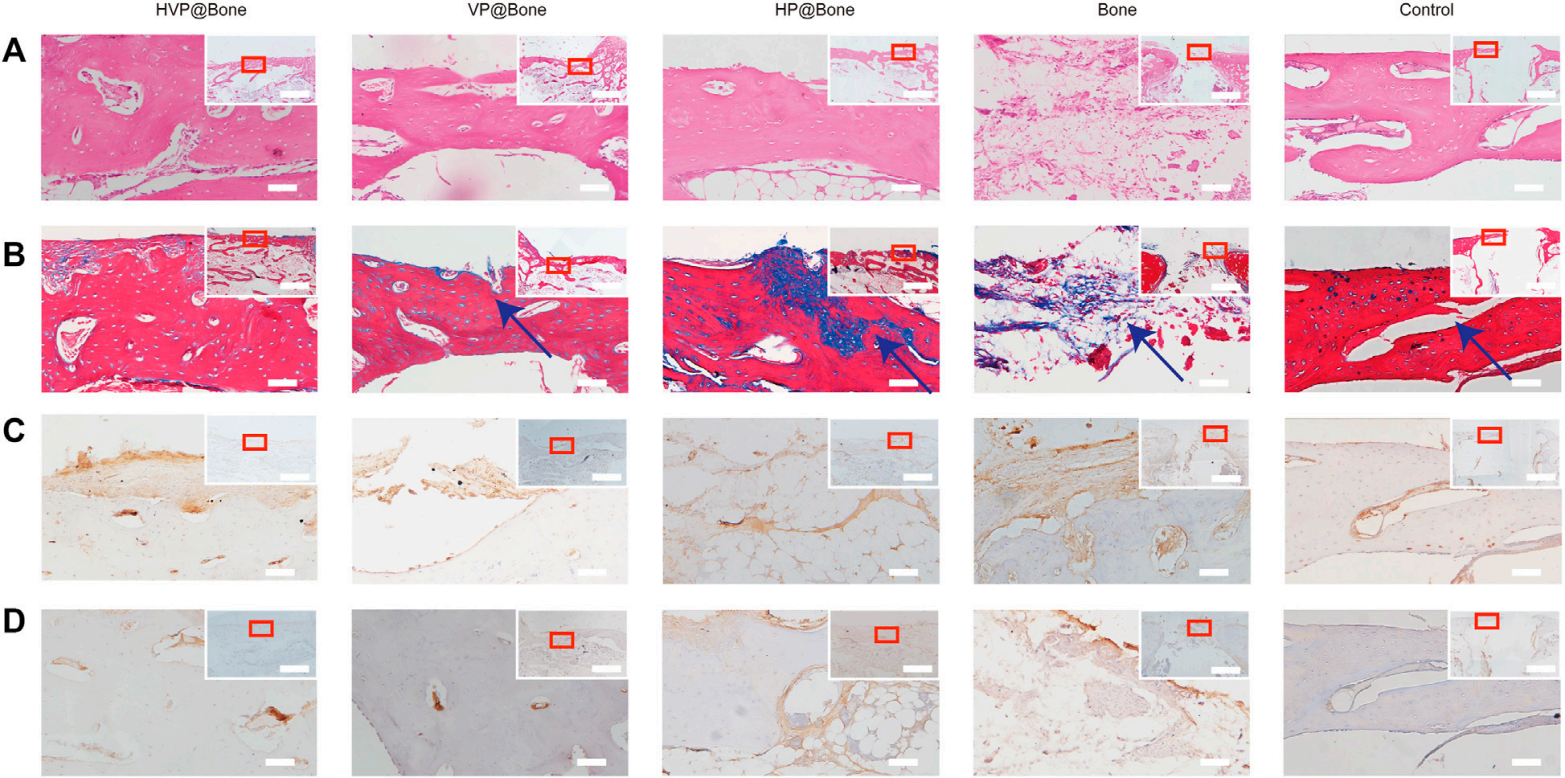
**(A)** Histological results of the H&E staining (200x mirror; scale bar: 50 μm). Histology and gross morphology at upper right corner (40x mirror; scale bar: 3 mm). **(B)** Histological results of the Masson’s trichrome staining (200x mirror; scale bar: 50 μm). Histology and gross morphology at upper right corner (40x mirror; scale bar: 3 mm). The defect area was marked with arrow. **(C)** Histological results of the type I collagen staining (200x mirror; scale bar: 50 μm). Histology and gross morphology at upper right corner (40x mirror; scale bar: 3 mm). **(D)** Histological results of the α-SAM staining (200x mirror; scale bar: 50 μm). Histology and gross morphology at upper right corner (40x mirror; scale bar: 3 mm).

After 12 weeks of implantation, the bone defects were repaired eminently by the HVP@Bone (Supplementary Figure S7). Obvious bone defects were visible in the control group. No infection or immune rejection was found in each group. The H&E staining results were consistent with the micro-CT reconstruction results. Compared with the control group, more bone cortex was observed in the HVP@Bone group, the VP@Bone group, and the HP@Bone group (Figure 6A). The HVP@Bone group showed complete bone cortex after 12 months. The osteoblasts were arranged orderly around internal and external circumferential lamella and collagen fibers in the intercellular matrix were arranged in layers. Incomplete and unsmooth bone cortex were observed in the VP@Bone group, the HP@Bone group and the Bone group. The osteoblasts were arranged irregularly and collagen fibers were arranged disorderly. No obvious regeneration was found in the control group. In the results of Masson’s trichrome staining, mature bone was stained red and regenerated collagen tissue of bone was stained blue. After 12 weeks of implantation, a completely regenerated bone cortex could be seen in the HVP@Bone group and was stained red (Figure 6B). Red mature bone and blue regenerated collagen fibers of could be seen at the defect in the VP@Bone group, the HP@Bone group and the Bone group. No obvious regeneration was found in the control group.

**FIGURE 7:**
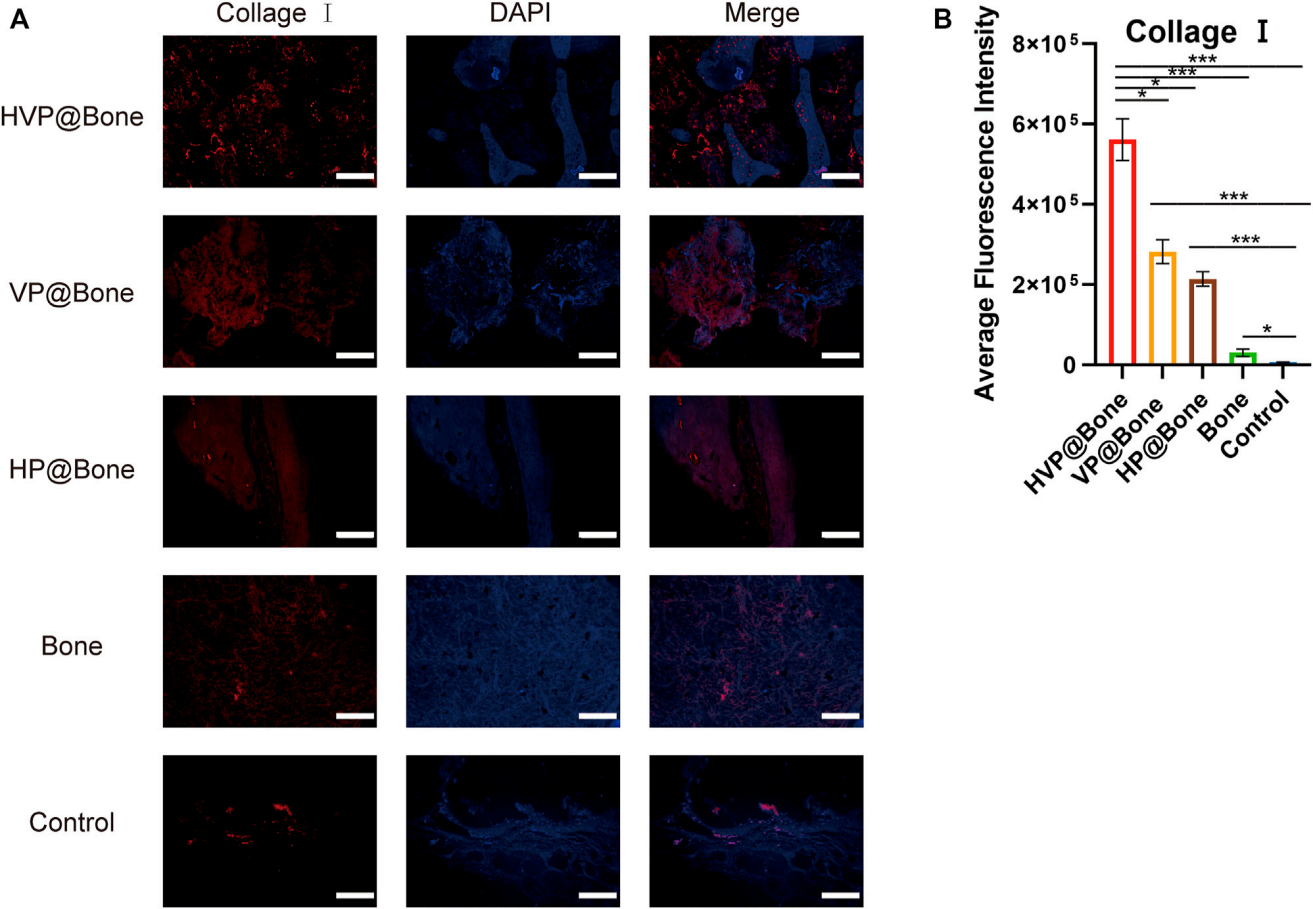
**(A)** Results of immunofluorescence staining of the type I collagen (scale bar: 200 μm). **(B)** Results of average fluorescence intensity of the type I collagen.

**FIGURE 8:**
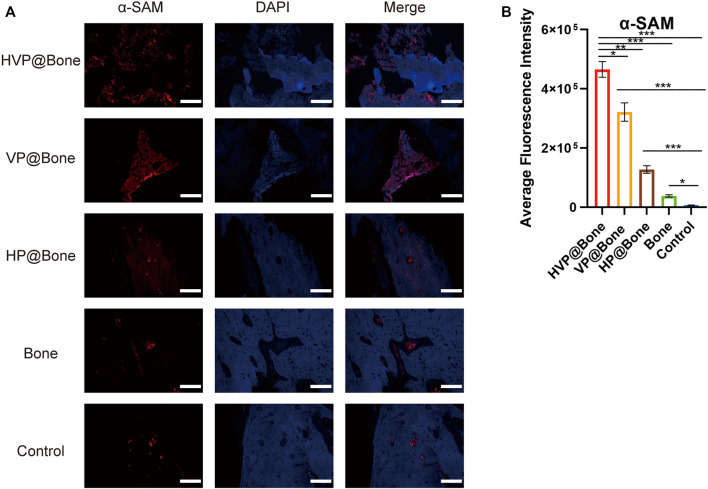
**(A)** Results of immunofluorescence staining of the α-SAM (scale bar: 200 μm). **(B)** Results of average fluorescence intensity of the α-SAM.

Immunohistochemical staining of type I collagen was performed to evaluate the bone regeneration in different groups. Strong collagen staining and neat cell arrangement were observed in the HVP@Bone group (Figure 6C). Compared with the HVP@Bone group, the color of the collagen was dimmed and disordered cell arrangement in the VP@Bone group, the HP@Bone group and the Bone group. There was no obvious collagen staining in the defect area of the control group. Immunohistochemical staining of α-SAM staining showed a great number of neovascularization could be seen in the repairing bone in HVP@Bone group and VP@Bone group. A small amount of neovascularization was seen in HP@Bone group and Bone group and no neovascularization was seen in the control group (Figure 6D). The same results were obtained by immunofluorescence staining of type I collagen and α-SAM staining. Obvious collagen staining was observed and more positive staining areas were seen around the defect in the HVP@Bone group, the VP@Bone group, the HP@Bone group and the Bone group. A few positive staining cells were seen around the defect in the control group. The image analysis software was used to further evaluate the number of positive staining for type I collagen and α-SAM. The positive staining area of the HVP@Bone group, the VP@Bone group, the HP@Bone group and the Bone group were significantly higher than the control group and the difference was statistically significant. The HVP@Bone group had more positive staining area than the Bone group (*p* < 0.05). The result demonstrated that the HVP@Bone group had exceptional bone regeneration ability among all of the groups (Figures 7, 8).

## Discussion

We employed PDA coating to attach VEGF on the surface of the allogeneic bone in this study. The surface morphology and composition of surface elements of VP@Bone are identical to that of allogeneic bone. *In vitro*, the HVP@Bone also displayed great biocompatibility and enhanced osteogenic signal expression. Meanwhile, it showed high osteogenic potential in a rabbit bone defect model compared with untreated allografts *in vivo*. Allograft bone was a popular option for reconstructive surgery because of its ready availability, favorable mechanical characteristics, and lack of donor-site morbidity. But allograft transplantation is said to have a 60% failure rate after 10 years after implantation because of fibrotic nonunions, infections, and secondary fractures (Wheeler and Enneking, 2005; Aladmawy et al., 2022).The following are the advantages of this study. 1) *In vivo* and *in vitro*, the HVP@Bone and VP@Bone showed good osteogenic and angiogenic effects compared with untreated allografts 2) The PDA adhesion method was straightforward and appropriate for therapeutic use. As a result, VP@Bone is an effective bone graft for bone regeneration and tissue vascularization, and VEGF adhesion with PDA coating is a simple and effective technique for allogeneic bone surface modification.

Bones may self-repair in most situations, but they may not heal on their own in conditions of large bone defects caused by trauma or bone tumor. Surgery was used to reconstruct bone defects and bone graft materials were used to promote bone repair. (Greenwald et al., 2001; Finkemeier, 2002; Faour et al., 2011; Campana et al., 2014). The application of bone regeneration therapy is quite promising at present and in the future. Allogeneic bone is a good bone graft material for the treatment of severe bone defect and nonunion. It provides mechanical support and good osteogenic and osteoconductive properties (Bow et al., 2019; Schmidt, 2021; ZhangY.B et al., 2021; Yang et al., 2022). However, Wisanuyotin *et al.* reported that allogeneic bone was used to reconstruct the bone defect after resection of primary bone tumors. Reconstruction failure in the allograft group was 55.3% (Wisanuyotin et al., 2022). The success rate of bone reconstruction may be increased by accelerating the vascularization of the bone tissue scaffold (Huang et al., 2022). Studies have shown that allogeneic bone combined with vascular growth factors promotes bone regeneration and increases osteoinduction ability (Schmidmaier et al., 2007).

Mussel adhesion proteins have good adhesion and biocompatibility, which makes it possible to modify the allogeneic bone surface with PDA coating (Sun et al., 2022). Combining allogeneic bone and growth factor is a viable option, according to the study and application of mussel adhesion protein (Kim et al., 2021). This was due to the copious reactive catechol groups created by PDA functionalization, which had a high affinity for the various nucleophiles (e.g., amines, thiols, and imidazoles) of peptides and proteins on cell surfaces. Meanwhile, the copious reactive catechol groups immobilized the mussel adhesion protein on the surface of allogeneic bone and formed the PDA coating. Tsai *et al.* reported that short-time dopamine incubation can enhance the adhesion of cells onto different materials (Tsai et al., 2011). Serum adhesion proteins were fixed by PDA coating on the surface of materials, thereby enhancing the adhesion of cells. As the result of Figure 2A–B, the adhesion and release of VEGF were not affected by short-time dopamine incubation. The adhesion efficiency of VEGF was 82.40% and approximately 68.80% of the total release of VEGF after 7 days. The VP@Bone produced a cell affinitive 3D microenvironment to encourage cell adhesion, spreading, and proliferation. As the result of SEM and EDS, PDA surface-modified allografts had a similar local surface microenvironment as untreated allografts. Early cell adhesion experiments revealed that more cells adhered to the allogeneic bone after PDA surface modification. This may be due to the high affinity of copious reactive catechol groups to cells. PDA had been shown to increase the recruitment and proliferation of bone progenitor cells (Hwang et al., 2010; Wu et al., 2022). Allogeneic bone with PDA surface modification promoted bone cell adhesion and proliferation. The adhesion of copious reactive catechol groups also made it possible to loading drugs, cells or other bioactive factors (Yin D et al., 2019; Ghorai et al., 2022; Wang et al., 2022). PDA surface modification of allogeneic bone was a straightforward, viable, and effective method.

VEGF was selected as a bioactive factor to promote angiogenesis and loaded on the surface of the allogeneic bone scaffold used in this study. Vascularized bone grafts accelerated bone remodeling and regeneration (Huang et al., 2022). VEGF (especially VEGF-A) is a powerful angiogenic factor that attracts endothelial cells to bone tissue and controls the differentiation and functioning of osteoblasts and osteoclasts, as well as engaging in bone remodeling (Zhang et al., 2023). The temporal-spatial signaling of VEGFA and PDGF-BB is integral to vessel maturation. VEGF with proper concentration can promote the migration and proliferation of endothelial cells and it may be related to the formation of type H vessels in bone (Peng et al., 2020). Previous research results showed type H vessels that are defined by high expression of CD31 and Endomucin (CD31hi Emcnhi) are associated with osteogenesis. Type H vessels are found near the metaphysis’ growth plate, as well as the diaphysis’ periosteum and endosteum. Type H arteries may be able to actively direct bone formation by producing substances that increase the proliferation and differentiation of osteoprogenitors in the bone marrow, according to evidence (Kusumbe et al., 2014). VEGF is attached to the surface of the allogeneic bone and gradually released after implantation. More VEGF adhered to the surface of PDA-coated allografts and released it more gradually, according to the VEGF dissolution release curve. The results *in vitro* demonstrated that VEGF-attached allogeneic bone increased the generation of osteogenic signal in the presence of endothelial cells.*In vitro* tests revealed that HVP@Bone had a greater osteogenic impact, which could be due to the following factors. 1) VEGF release promotes the mobilization and recruitment of endothelial (progenitor) cells, which helps to speed up the vascularization of bone tissue. (Huang et al., 2022). 2) In addition to sticking to VEGF, PDA coating can cling to bone progenitor cells, which promotes bone progenitor cell adhesion and reproduction (Wu et al., 2022; Zhang et al., 2022). These factors of this paper are extremely important in clinical application and it is of great significance for clinical healing of bone defects. At present, the failure rate of bone transplantation is 22% in the treatment of some large long shaft defects (Feltri et al., 2022). The main reason lies in the failure to rebuild the blood supply of the defect in time and the accumulation of bone progenitor cells. The application of this scheme can accelerate the vascularization of bone defects, which is conducive to the aggregation and regeneration of bone progenitor cells, to accelerate bone reconstruction. Therefore, PDA-coated allogeneic bone with VEGF is an effective surface modification method to promote bone regeneration.

There are a few limitations to the current study that should be mentioned. In this study, specific osteogenic and angiogenic pathways were not validated. There is a link between osteogenesis and angiogenesis, and the specific pathway should be investigated further in the studies to follow. Furthermore, the rate at which VEGF is released should be regulated. The development of functioning blood vessels is aided by an optimum VEGF release rate. The binding of the regulated VEGF release platform to allogeneic bone is an area that should be investigated further.

## Conclusion

In summary, the allogeneic bone can be modified with PDA coating under simple conditions and effective adhesion of VEGF. The surface modified allogeneic bone has a familiar surface structure and element content with natural allogeneic bone. Moreover, the HVP@Bone has better biological and can release VEGF continuously and stably. The HVP@Bone can increase the expression of signals relevant to osteogenesis *in vitro* and promote bone regeneration and angiogenesis *in vivo*. DOPA surface modification is a simple and effective way to enhance the surface adhesion of allogeneic bone, and can enhance the effect of osteogenesis and angiogenesis by adhering VEGF.

## Data Availability

The original contributions presented in the study are included in the article/Supplementary Material, further inquiries can be directed to the corresponding authors.
