# Supplementary material for: Covalent immobilization of VEGF on allogeneic bone through polydopamine coating to improve bone regeneration
Source: Front Bioeng Biotechnol. 2022 Oct 12;10:1003677. doi: 10.3389/fbioe.2022.1003677 (PMC9597090; doi:10.3389/fbioe.2022.1003677)
Supplement: Supplementary file 1 [file DataSheet1.docx]

**COVALENT IMMOBILIZATION OF VEGF ON ALLOGENEIC BONE THROUGH POLYDOPAMINE COATING TO IMPROVE BONE REGENERATION**

Jianhao Huang^1#^, Jingwei Lu^2#^, Ziying Liu^2^, Jing jin^3^, Chunmei Xie^4^, Yang Zheng^5^, Zhen Wang^2^, Lingfeng Yu^2^, Yan Zhu^2^, Gentao Fan^2^, Guojing Sun^2^, Zhihong Xu^3*^, Guangxin Zhou^1,2*^

^1^Department of Orthopedics, Jinling Hospital, the first School of Clinical Medicine, Southern Medical University, Nanjing 210002, China.

^2^Department of Orthopedics, Jinling Hospital, Nanjing University, Nanjing 210002, China.

^3^State Key Laboratory of Pharmaceutical Biotechnology, Division of Sports Medicine and Adult Reconstructive Surgery, Department of Orthopedic Surgery, Drum Tower Hospital affiliated to Medical School of Nanjing University, Nanjing, 210000, China.

^4^Hangzhou Lancet Robotics Company Ltd, Hangzhou, 310000, China.

^5^Nanjing Yaho Dental Clinic, Nanjing, 210000, China

**Figures:**

**
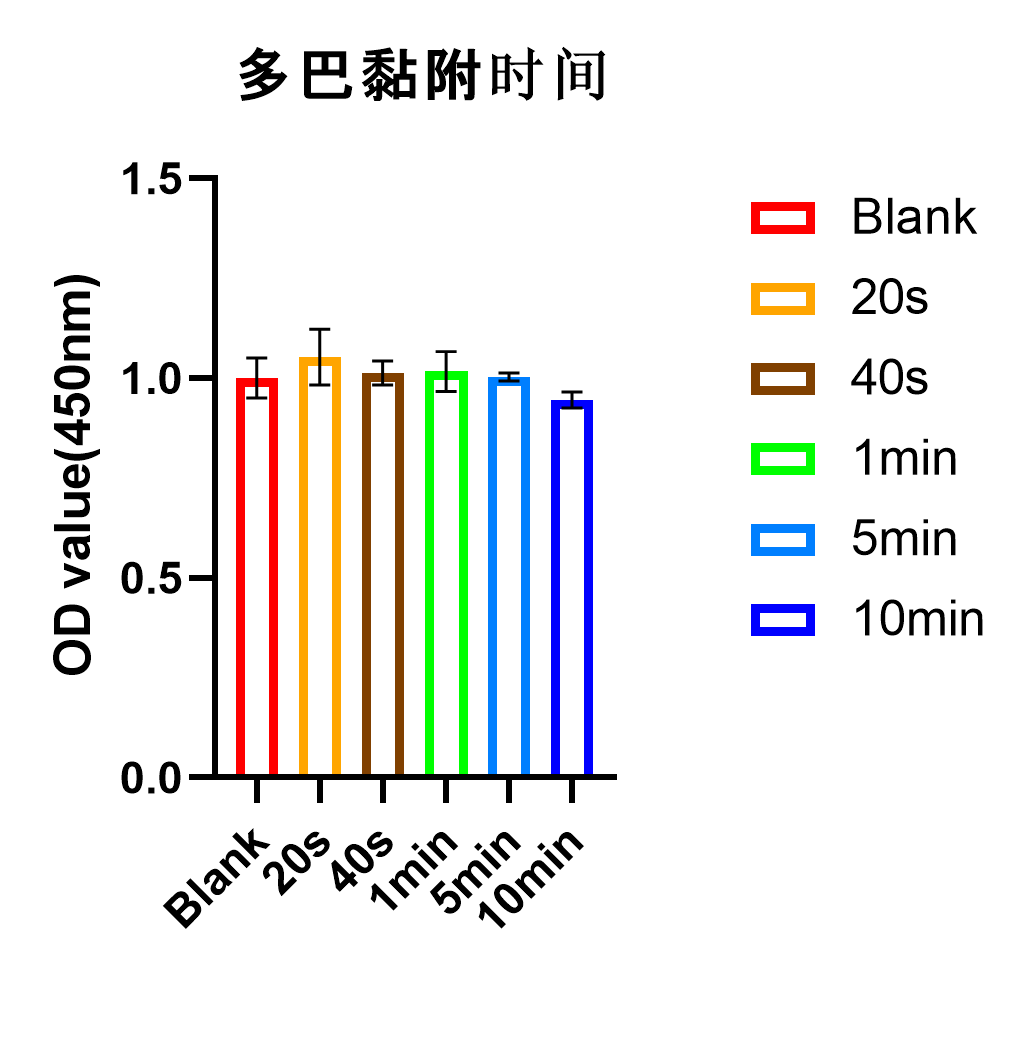
**

**Figure S1** The relative cell vitality after culturing 72h with DOPA at different points in time.


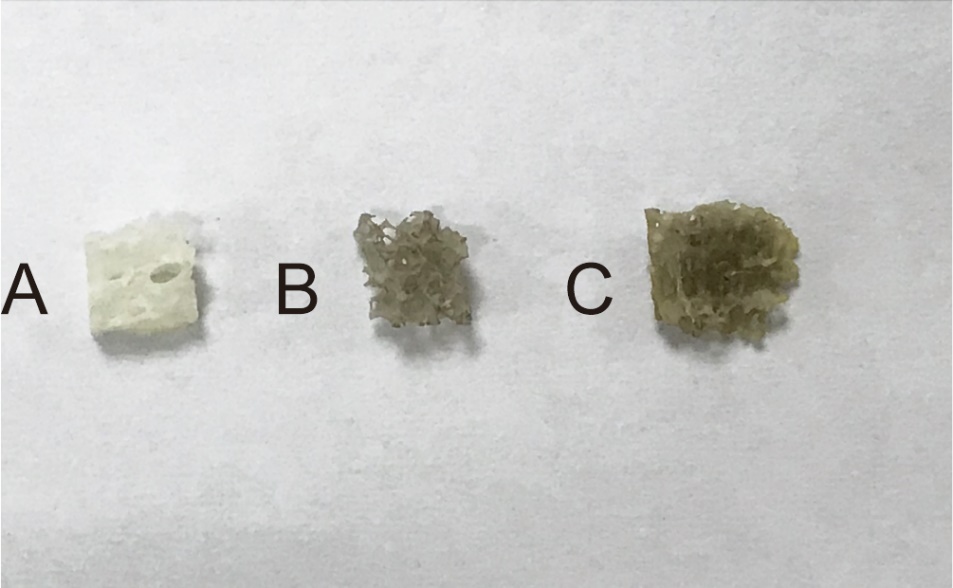


**Figure S2** General view of untreated allogeneic bone(A), P@Bone (B) and VP@Bone(C).


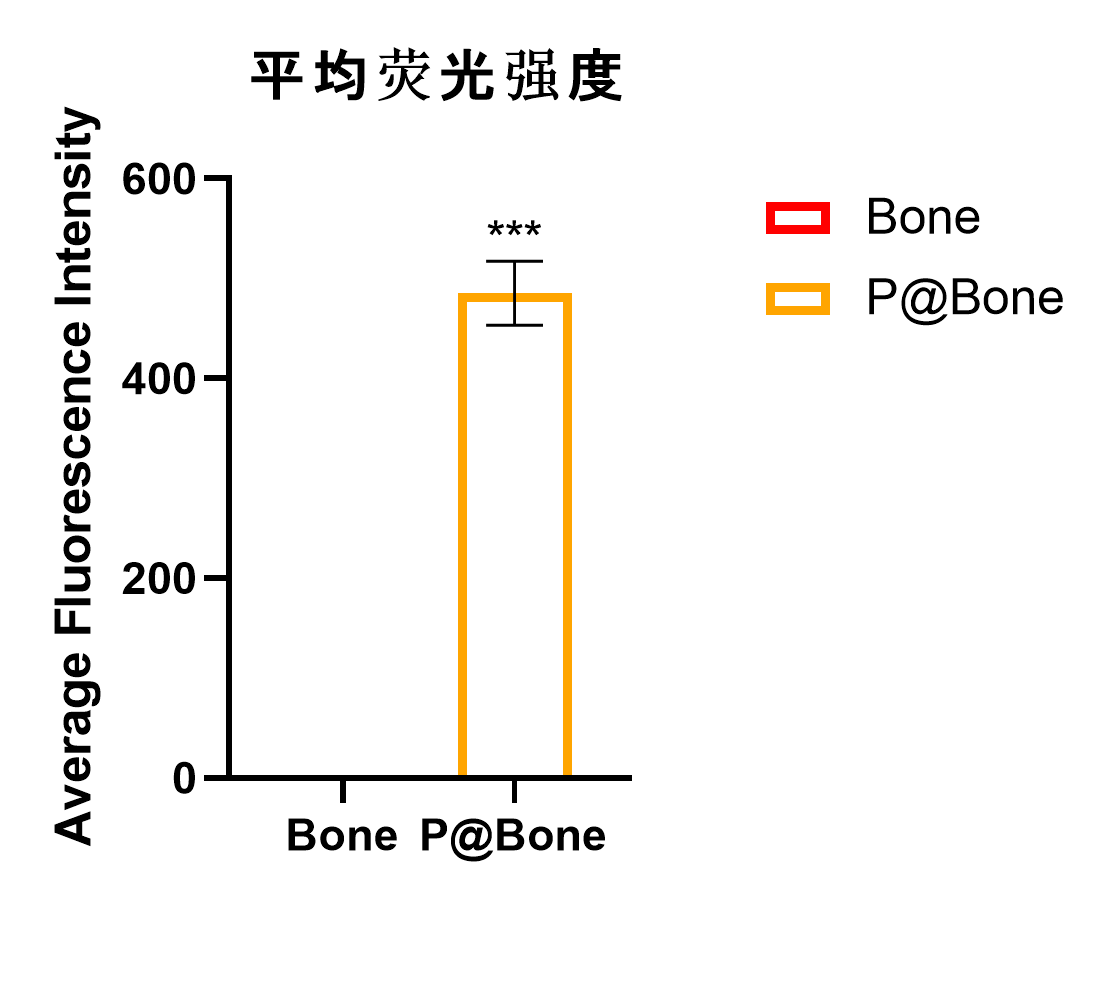


**Figure S3** The average fluorescence intensity of the Phalloidine/DAPI staining in early adhesion experiments.


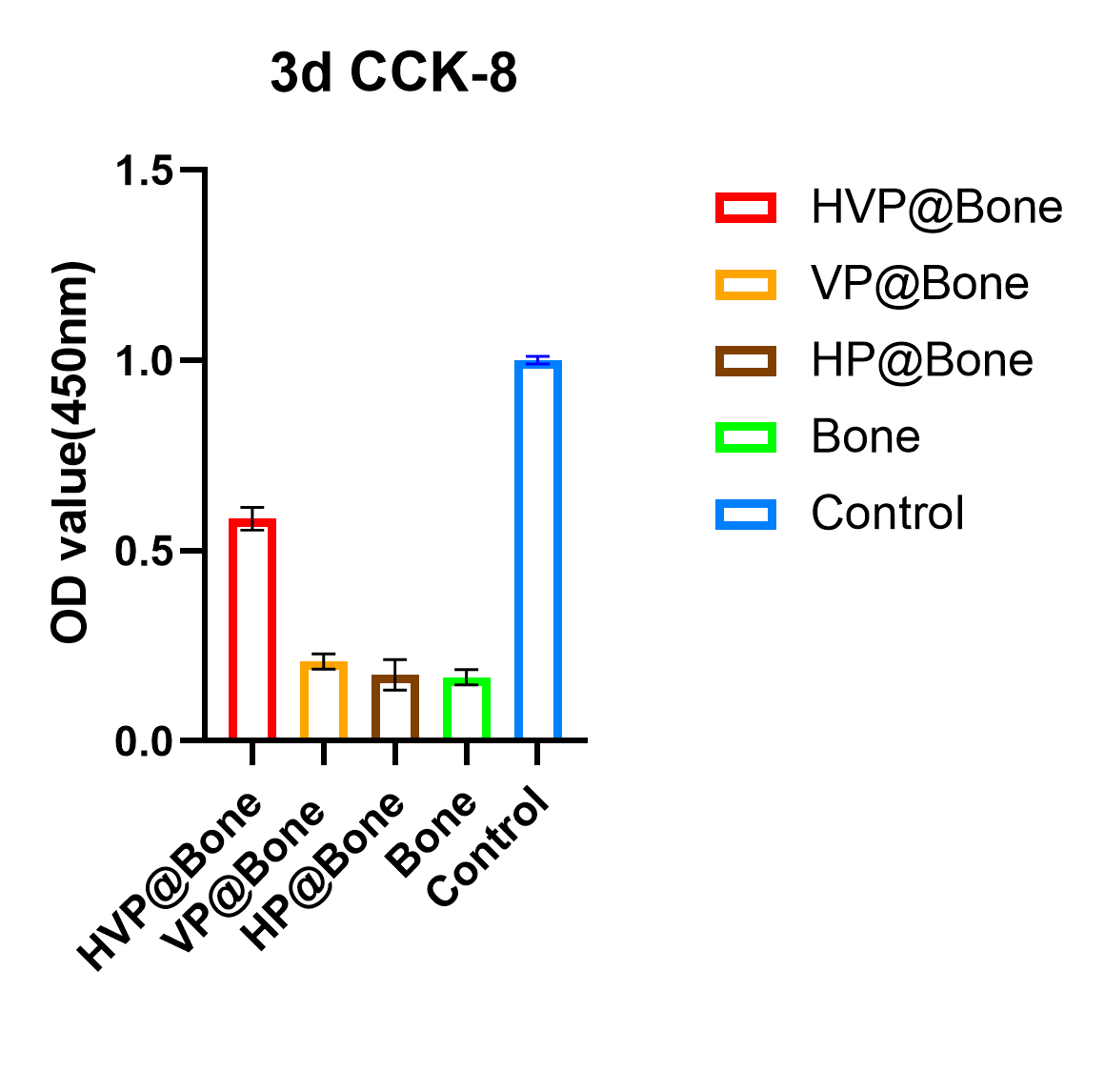


**Figure S4** The relative cell vitality after culturing for 3 d.


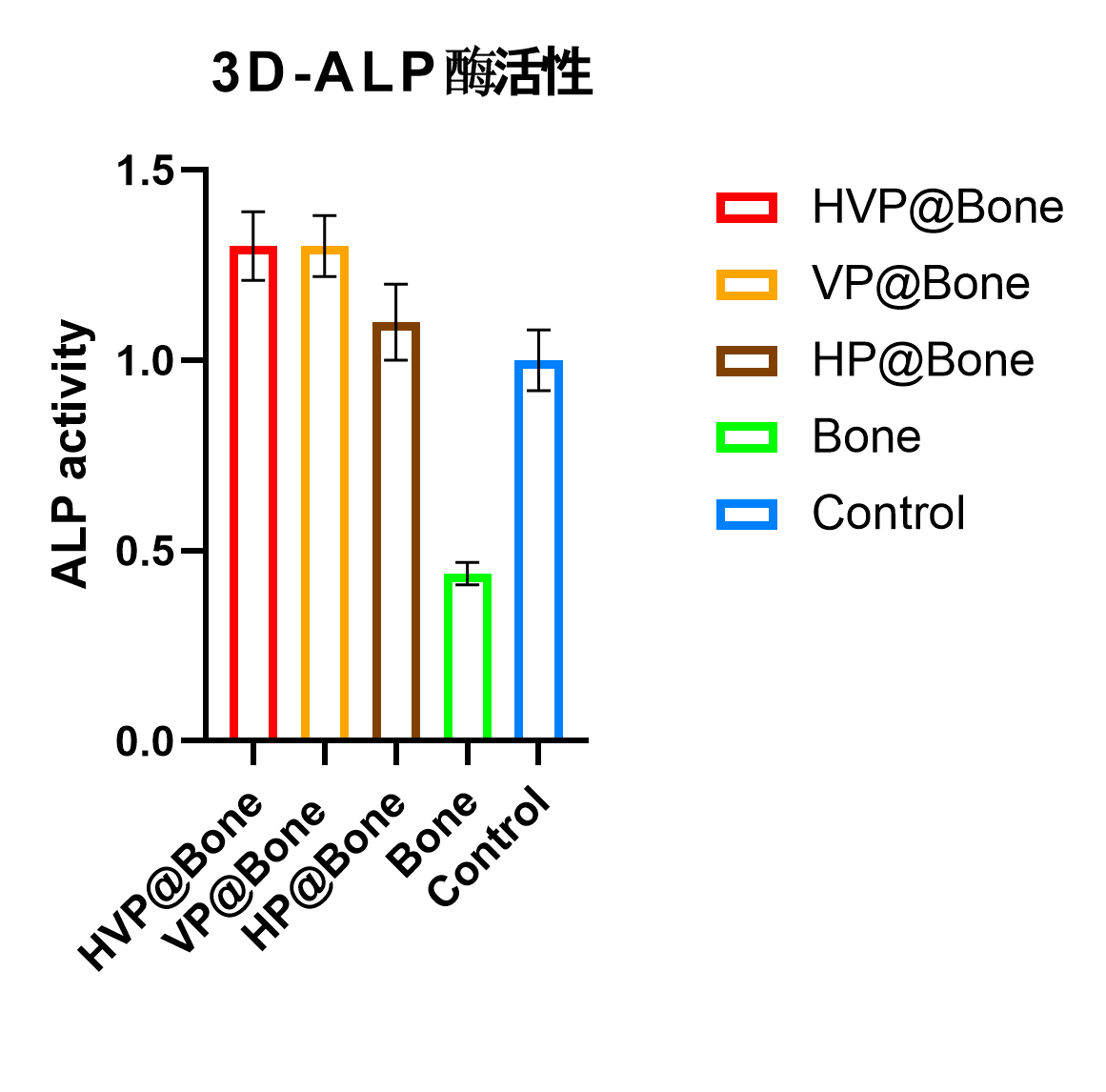


**Figure S5** The culture medium ALP activity at 3 days.


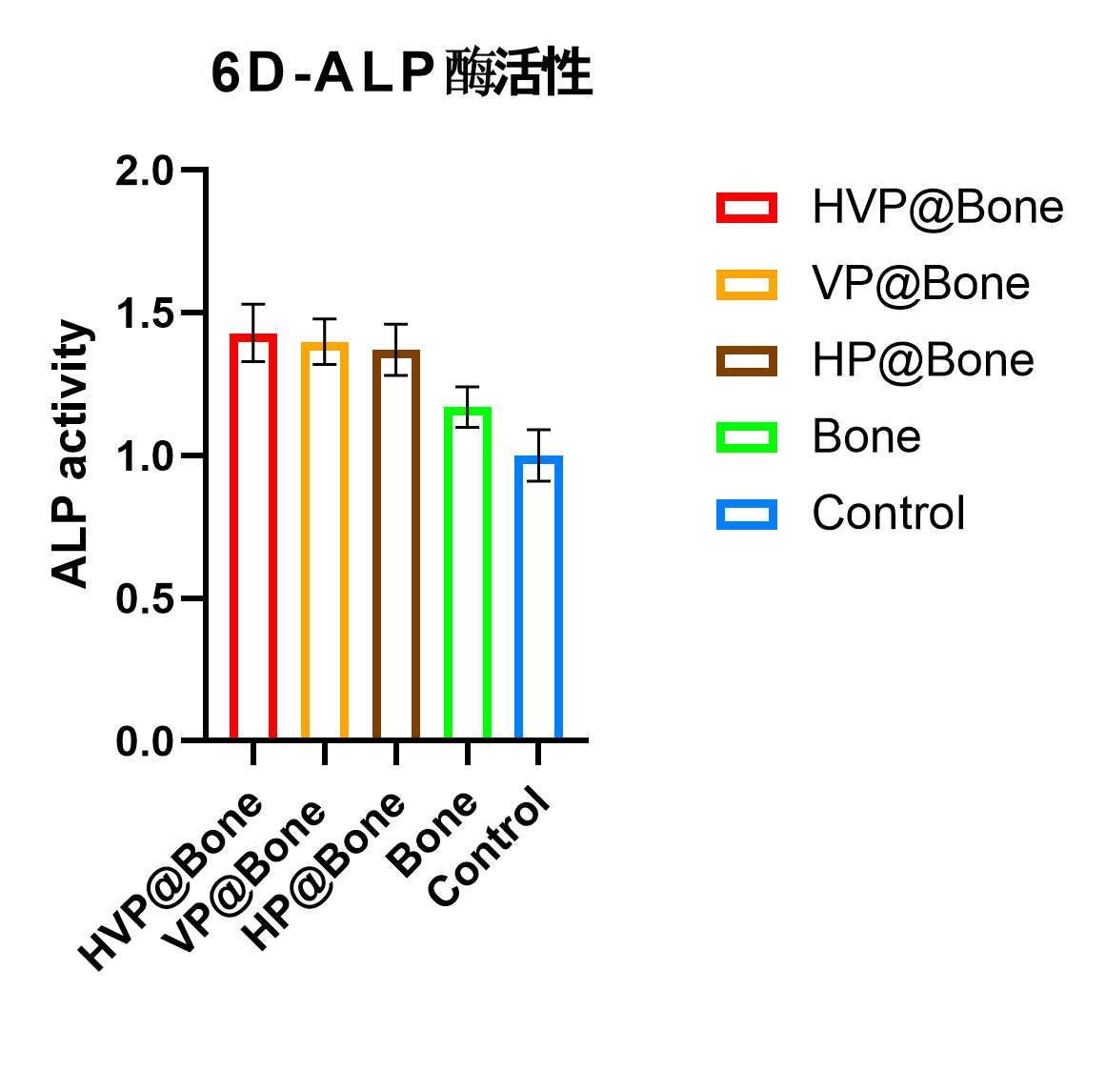


**Figure S6** The culture medium ALP activity at 6 days.


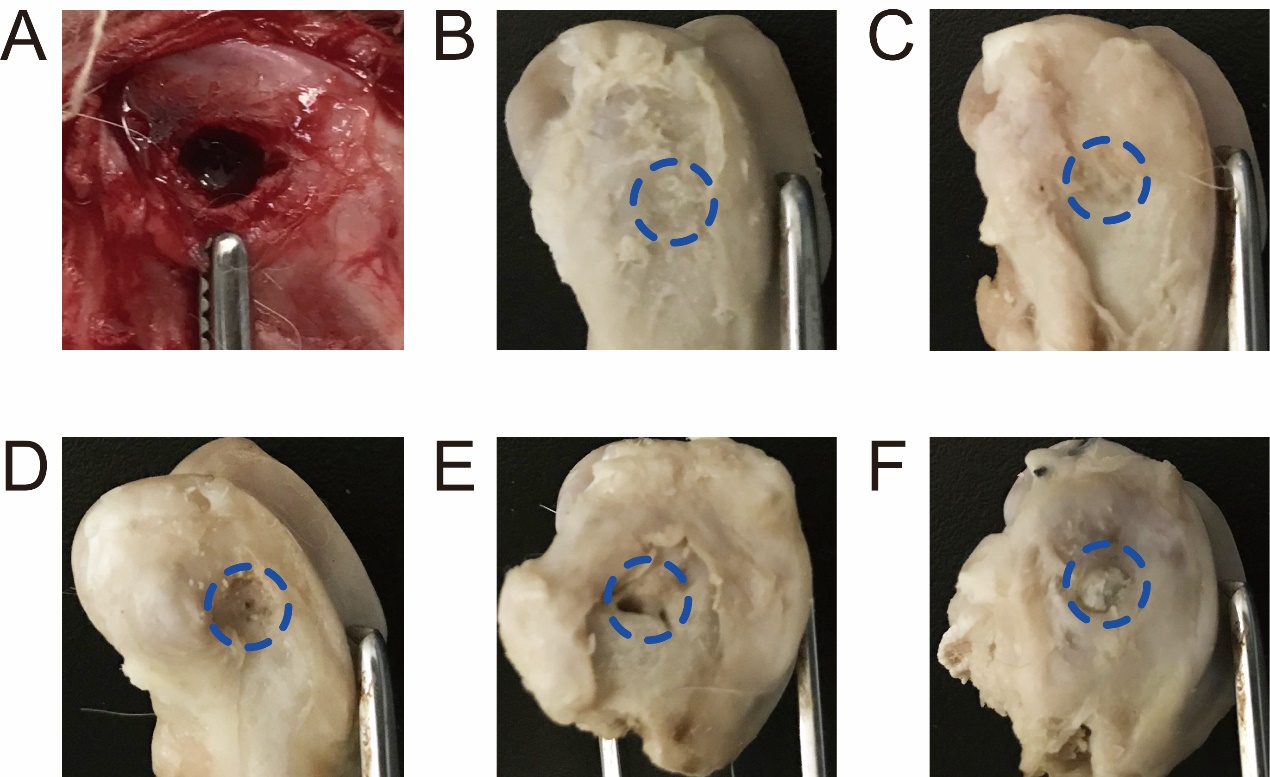


**Figure S7** (A) General view of femoral bone defect model. (B-F) General view of HVP@Bone group, VP@Bone group, HP@Bone group, Bone group and control group after 12 weeks of operation.

**Table S1** Corresponding RT-qPCR Primers

|  | forward | reverse |
| --- | --- | --- |
| GAPDH | 5′‐GGAGCGAGACCCCACTAACATC‐3′ | 5′‐CTCGTGGTTCACACCCATCAC‐3′ |
| Collage Ⅰ | 5′‐GGCAAAGATGGAGAAGCTGG 3′ | 5′‐GGAAACCTCTCTCGCCTCTT3′ |
| OCN | 5′‐GGACCATCTTTCTGCTCACTC3′ | 5′‐CTGCTTGGACATGAAGGCTT3′ |
| Runx-2 | 5′‐CCAGATGGGACTGTGGTTACC‐3′ | 5′‐ACTTGGTGCAGAGTTCAGGG‐3′ |
